# Estimating the Heterogeneous Relationship between Peer Drinking and Youth Alcohol Consumption in Chile Using Propensity Score Stratification

**DOI:** 10.3390/ijerph111111879

**Published:** 2014-11-17

**Authors:** Yoonsun Han, Andrew Grogan-Kaylor, Jorge Delva, Yu Xie

**Affiliations:** 1 Department of Child Psychology and Education, Sungkyunkwan University, 25-2 Sungkyunkwan-ro, Jongno-gu, Seoul 110-745, Korea; 2 School of Social Work, University of Michigan, 1080 South University Avenue, Ann Arbor, MI 48109, USA; E-Mails: agrogan@umich.edu (A.G.-K.); jdelva@umich.edu (J.D.); 3 Institute for Social Research, University of Michigan, 426 Thompson Street, Ann Arbor, MI 48104, USA; E-Mail: yuxie@umich.edu

**Keywords:** adolescent behavioral health, Chile, peer alcohol consumption, propensity score stratification

## Abstract

When estimating the association between peer and youth alcohol consumption, it is critical to account for possible differential levels of response to peer socialization processes across youth, in addition to variability in individual, family, and social factors. Failure to account for intrinsic differences in youth’s response to peers may pose a threat of selection bias. To address this issue, we used a propensity score stratification method to examine whether the size of the association between peer and youth drinking is contingent upon differential predicted probabilities of associating with alcohol-consuming friends. Analyzing a Chilean youth sample (*N* = 914) of substance use, we found that youths are susceptible to the detrimental role of peer drinkers, but the harmful relationship with one’s own drinking behavior may be exacerbated among youth who already have a high probability of socializing with peers who drink. In other words, computing a single weighted-average estimate for peer drinking would have underestimated the detrimental role of peers, particularly among at-risk youths, and overestimated the role of drinking peers among youths who are less susceptible to peer socialization processes. Heterogeneous patterns in the association between peer and youth drinking may shed light on social policies that target at-risk youths.

## 1. Introduction

Peer relationships have long been documented as a key factor in explaining youth drug-use and delinquency [1]. This is not surprising given that adolescence is a phase of the life course in which intimate peer relationships start to develop and are strengthened [2,3]. An increased amount of time spent with peers, as well as enhanced quality of peer interactions based on a better understanding of others’ thoughts and feelings, allow such relationships to emerge during this developmental stage. Beyond intimate friendships, there is also a broader category of peer groups that construct impersonal, yet powerful, norms of youth behavior [4]. Whether it is through intimate interactions or overarching subculture, peers increasingly have a growing influence over youth’s behavior and beliefs from early adolescence. As an example of the socialization process, peers may affect the individual by exerting an influence on perceptions and on aspects of socio-moral reasoning, such as beliefs, values, and standards [1]. Furthermore, adolescents are socialized by an array of peers; from those who provide a reference point to those who exert pressure for conformity to peer standards [5].

Peer socialization is particularly salient in alcohol consumption behavior during adolescence. Alcohol consumption by close friends or peer groups has been identified as a possible trigger that may lead to the onset of youth drinking, and can encourage intoxicating levels of consumption through mechanisms of peer modeling and shared norms [6,7,8,9]. In the context of peer relationships and adolescent drinking behavior, however, the inherent heterogeneous nature of individuals, which inevitably generates different levels of peer interaction across youth, has received relatively less attention. It has been well documented that youth’s involvement with drinking peers serves as a mechanism that reinforces youths’ own use of alcohol [6]. However, it has also been empirically observed that some youth are more affected by the presence of alcohol-drinking peers than others, despite similar levels of exposure to delinquent peers, even after controlling for background characteristics [10]. Individual heterogeneity in levels of self-regulation [10], self-esteem and positive affectability [11], and aggression [12] may serve as possible explanations for resilience against, or vulnerability towards, the influence of peer drinking behavior.

In sum, when identifying the youth-peer relationship in substance use, it is important to control for a wide range of variability in individual, family, and social factors. Further, it is crucial to note that adolescents differ greatly not only in terms of background characteristics, but also in how adolescents uniquely respond to these factors (a.k.a., “heterogeneous treatment effects”). Substantively then, neglecting to acknowledge heterogeneous relationships may imply making an unrealistic typological assumption that all youth respond to exogenous factors homogenously. Methodologically, failure to account for the pervasiveness of intrinsic differences in youth’s response to peers may generate bias in estimating the youth-peer association in alcohol use.

### 1.1. An Alternative Statistical Approach: Propensity Score Stratification

The variability in the response to peer socialization processes across youth has commonly been overlooked in most observational studies on this topic. This study takes an alternative approach to examining the complexity of peer relationships and alcohol consumption behavior. We investigated the possibility of different peer-youth associations across groups of youth with systematically different characteristics using a propensity score stratification method.

#### 1.1.1. Statistical Explanation

A propensity score is the conditional probability of being assigned to a treatment when controlling for observed variables [13]. The propensity score contains rich information on a variety of observed background characteristics, which is distilled to a single number [14]. In randomized experiments, the propensity score is equal across individuals, such that every participant in the study has an equally random chance of receiving the treatment. In nonrandomized observational studies, however, this is typically not the case [13].

Propensity score stratification may partially control for systematic differences, by stratifying individuals into subclasses based on a propensity estimated according to the observed variables [13]. By sub-classifying individuals into groups of similar propensities, and assuming homogeneity at the group level, we can compute treatment effects within those groups. We may rely on stratification across groups because in the real world it is impossible to compute individual relationship sizes since the counterfactual of a behavioral or social event can never be observed [15]. Therefore, relationship parameters of interest can only be computed at the group level under some homogeneity assumption within groups, which is a feasible improvement in estimating treatment effects over not allowing for any variation at all [15]. When the balancing condition is satisfied for each subclass, treatment and control groups within these subclasses can be directly compared. In this context, balancing implies that between the treatment and control groups within each stratum: (a) the conditional distribution of observed covariates and (b) the average propensity score are not statistically different [16,17]. Thus, when the balancing properties are satisfied, despite different distributions between treatment and control groups overall, the distributional properties are similar between the two groups within each of the stratified subgroups. As such, propensity score stratification attempts to establish a framework similar to a randomized experiment in which direct comparison between the treatment and control groups is possible. Propensity scores can be a particularly useful analytical tool for examining the effect of interpersonal relationships within a broader social context (e.g., adolescent peer relationships) in comparison to the treatment effect that needs to be estimated in isolation through an experimental design.

However, the propensity score stratification method is not without limitations. Although a five-group propensity score stratification method has been found to improve much of the bias from observed variables, the distributional balance of *unobserved* characteristics is not always achieved [17]. Only when invoking the assumption of strongly ignorable treatment assignment (*i.e.*, after controlling for observed variables, there are no confounding variables that link the outcome and treatment), are we able to derive unbiased estimates of the average treatment effect for each stratum [13]. The strongly ignorable treatment assignment assumption, however, is an unverifiable assumption. Nevertheless, employing a comprehensive set of control variables in constructing the propensity score has been found to make the strongly ignorable treatment assignment assumption more plausible. Furthermore, conducting sensitivity or auxiliary analyses are alternative methods to assess the plausibility of the assumption [16].

#### 1.1.2. Application

Propensity score stratification methods can help our understanding of adolescent peer relationships and alcohol consumption behavior. To date, most studies have estimated a single measure for the relationship between peer drinking and youth drinking [18,19,20]. Computing a single relationship measure implies assuming that all youth respond to peers equally. In statistical terms, the coefficient of the treatment effect (δ), which is the association with drinking peers (*d_i_* = 1) or not (*d_i_* = 0), is a constant parameter that is equal across all individuals (*i* = 1, 2, …, *n*). A growing body of literature, however, has suggested that there is individual heterogeneity in peer-youth relationships [21]. In other words, some youth are at greater risk for developing antisocial behavior through peer influence than others, and therefore, the assumption that all youth will respond identically to peer socialization may not be appropriate. When assuming variation in the response to the treatment across individuals, we can attach the subscript *i* to the treatment effect (δ*_i_*).

The underlying assumption for investigating heterogeneous treatment effects is that differences in vulnerability to developing pathological peer relationships may determine the level of influence that antisocial peers have on inducing youth’s own antisocial behavior [10]. Due to unique demographics, family backgrounds, social factors, and personality and behavioral traits, youth do not have an identical chance of affiliating with problem peers [22,23,24]. In other words, the role of peers may be systematically different between individuals who are more susceptible to having antisocial friends and those who are less susceptible. Not accounting for such heterogeneity in the size of the relationship between peer and youth substance use across individuals may be a threat to estimating unbiased measures, particularly in observational studies.

### 1.2. Hypothesis and Analytic Strategy

Based on this evidence, we employed a five-step analytic strategy using propensity score stratification to identify heterogeneous patterns in the relationship between peer drinking and youth drinking. First, using probit regression, we computed propensity scores by estimating an individual’s predicted probability of associating with friends who drink, based on observed information. Propensity scores can be computed to stratify youth based on observed characteristics, such as individual, family, and social contextual factors, all of which are risk factors for alcohol consumption [11]. In our study, the propensity score was the conditional probability of associating with peers who drink:

(1) $$\text{Pr}\left(d_{i}=1|X_{i}\right)=\text{Φ}\left(\overset{K}{\underset{k=0}{\sum }}\beta_{k}X_{ik}\right)$$

In the propensity score equation, *X_i_* is a vector of *K* number of observed measures that predict the probability of associating with drinking peers for each youth (*i*). β denotes a set of coefficients that estimates the relationship between the covariates and the probability of friends being drinkers, under the cumulative normal distribution (Φ). Second, we stratified our sample into five groups based on these propensity scores and checked for the balance conditions. We invoked the strongly ignorable treatment assignment assumption, so that within each stratum, youth who affiliated with peers who drink and youth who do not have any affiliation with peers who drink were balanced. We achieved the balancing condition within each stratum by modifying the propensity score model with higher-order and interaction terms of covariates predicting the propensity score [25]. Third, for each stratum, we estimated the relationship between peer drinking and youth drinking using a negative binomial regression count model (Level-1):

(2) $$\text{log}\mu_{is}=\alpha_{s}+\delta_{s}d_{is}$$

In the present study, the *i*^th^ individual is nested in each stratum (*s* = 1, 2, …, *S*). The outcome, μ_is_ is a count variable that represents the number of drinks consumed in the past 30 days for each stratum. The parameter, δ*_s_*, represents heterogeneous treatment effects across strata, and α*_s_* is a constant. Fourth, we investigated heterogeneous peer-drinking effects across strata using a hierarchical linear model (Level-2):

(3) $$\delta_{s}={\hat{\delta }}_{1}+\gamma S+\upsilon_{s}$$

Using a linear regression model, we regressed the Level-1 slopes (δ*_s_*) on the propensity score strata (*S*) to identify a linear trend in the treatment effects (γ). $\hat{\delta }$ is the Level-2 intercept and υ*_s_* is the residual. Results from a hierarchical linear model that identifies the variation of relationships across stratum may provide greater support for the existence of heterogeneity in the relationship between peer-drinking and youth-drinking patterns. Fifth, we conducted auxiliary analyses to check whether it was reasonable to invoke the strongly ignorable treatment assumption when interpreting our findings.

### 1.3. Contribution

Although differential response to treatment is the norm in human interactions, there have been very few studies that investigate heterogeneous treatment effects in the social and behavioral sciences [16,26]. Most studies using observational data have assumed homogeneous relationship sizes when estimating the association between peer and youth’s own antisocial behavior; this exploratory study differs from other substance use research in this respect. To our knowledge, all studies of alcohol use among youth in Chile using cross-sectional designs have focused on obtaining prevalence estimates or computing associations, without addressing the potential influence of unobserved individual heterogeneity [27,28]. Therefore, our propensity score analytic approach not only contributes to a better understanding of associations between peer and youth drinking by employing innovative and advanced methodological techniques, but, in using a Chilean sample, we may address the growing interest and demand for cross-cultural studies [29].

## 2. Method

### 2.1. Data

Data in the present study were obtained from the Santiago Longitudinal Study (SLS), which examines substance use among adolescents from neighborhoods of low- to mid-socioeconomic status in Santiago, Chile. The SLS is a joint project between Chilean and U.S. institutions and received funding from the U.S. National Institute on Drug Abuse (NIDA). Youth participants were recruited from a sample of 1200 youth who had participated in a study of iron and nutritional status at the Institute of Nutrition and Food Technology, University of Chile when they were 10 years old [30]. In the period 2007–2010, 1076 youth ages 12–17 years old completed a comprehensive assessment of substance using behaviors. For the present study, we used data from the 914 (84.9%) observations that contained full data for the variables of interest. Results from auxiliary analyses using mean difference tests on key variables of the study (*t*-test for continuous variables and chi-square test for categorical variables) indicated that missing data occurred at random, and hence omitting the data from our analysis would not have had an impact on the interpretation of our main results.

### 2.2. Measures

The independent variable and all the control variables were selected on a theoretical and empirical basis to predict the dependent variable and propensity score. The comprehensive set of measures included dummies and square values to improve the balance on the observed variables [14,17]. The functional form of covariates was chosen based on multiple tests of fit and stepwise procedures.

#### 2.2.1. Dependent Variable

The dependent variable was the estimate of the *total number of drinks* consumed in the past 30 days. It was the product of the number of days consumed in the past 30 days and the average number of drinks consumed each day.

#### 2.2.2. Independent and Control Variables

The independent variable was a binary variable of whether the *youth associated with friends who consumed alcohol* (*d_i_* = 1) or did not associate with any drinking friends (*d_i_* = 0) in the past 30 days. Control variables included youth demographics, parent and family characteristics, and social contextual factors. All control variables in the main model were also used to predict the propensity score. Specifically, *male* adolescents were coded as 1 and females as 0, and *age* was measured as the age at the time of the interview. As for family and parent variables, household *income* was measured in Chilean pesos (500 pesos = ~1 US$). Parents’ level of *completed education* was categorized into four groups—less than middle school, middle school to less than high school, high school, and some college or more. Parents’ *marital status* was coded as 1 if married at the time of the interview and 0 if otherwise. Parents’ level of *alcohol consumption* was the frequency of alcohol consumed by both parents (one parent if the youth was from a single-parent household) in the past 30 days. Three items on a five point scale (“never” = 1, “very often” = 5), which asked about perceived levels of neighborhood crime (e.g., muggings, burglaries, assaults) and drug sales and use, were combined into one measure of *neighborhood danger and drugs* (α = 0.71). Youth’s *exposure to alcohol advertisements* on television and magazines/newspapers were each categorized into three dummy variables (“0–1 times/month,” “1–9 times/month,” or “1 or more times/day”).

#### 2.2.3. Auxiliary Variables

Finally, we utilized several youth behavioral measures to assess the plausibility of invoking the ignorability assumption in our analysis. We examined the following dispositions as they have been identified in the literature to be associated with greater susceptibility to negative peer influence [1]. Auxiliary variables were not incorporated in the propensity score as it is difficult to consider them as pretreatment covariates. *Rule-breaking* (e.g., “I do not feel guilty after misbehaving,” “I break rules at home, school, or elsewhere) and *aggression* (e.g., “I am mean to others,” “I destroy my own things”) were the sum of 15 (α = 0.69) and 17 (α = 0.81) items, respectively, from the Youth Self Report [31]. The Youth Self Report contains 112 items used to assess the youth’s behavioral status in the past 6 months. Youth were asked to indicate if the items were “not true” = 0, “somewhat or sometimes true” = 1, or “very true or often true” = 2. *Risk-taking* behavior measured the level of risky daily activities including gang involvement, reckless driving, and sexual activity in the past week [32]. It was measured using 13 items (α = 0.72) with five response categories (e.g., “never” = 1, “this past week” = 5). Finally, *self-esteem*, a measurement of social acceptance, confidence, and life satisfaction was also examined [32]. Nine items (α = 0.82) assessed the degree to which statements about positive traits correctly described the youth, and each item had five response categories (e.g., “do not agree” = 1, “completely agree” = 5).

## 3. Results and Discussion

### 3.1. Descriptive Analysis

Table 1 reports the descriptive statistics of the sample for youth who had friends who drank (*N* = 631) and those who did not (*N* = 283). The average number of drinks consumed within the past 30 days was greater for individuals who affiliated with drinking peers than that of individuals who did not have a drinking friend. Youths who associated with peer drinkers mostly came from families of somewhat higher levels of socioeconomic status. Broader contextual factors of neighborhood conditions also distinguished youth who had affiliations with peer drinkers. Greater levels of danger and drug deals in neighborhoods, and greater exposure to alcohol advertisements in newspapers, magazines, and on television were reported among individuals who had friends who drink, compared to those who did not. In sum, the descriptive statistics suggest that a broad array of observed factors were related to associating with peers who drink.

**Table 1:** Descriptive Statistics of Youth by Peer-Drinking Status.

Variable	(1) Have Friends Who Drink (*n* = 631)		(2) No Drinking Friends (*n* = 283)		*p*-Value ^a^
	Mean (%)	Standard Deviation	Mean (%)	Standard Deviation	
*Youth*					
Number of drinks consumed in the past 30 days	2.27	7.90	0.07	0.29	<0.001
Male	50%	--	54%	--	0.178
Age	14.79	1.47	13.49	1.05	<0.001
*Family Context*					
Monthly income (unit: 100 Chilean pesos)	3.19	1.60	2.98	1.45	0.056
Less than middle school	6%	--	6%	--	0.932
Middle school to less than high school	35%	--	43%	--	0.013
High school	56%	--	47%	--	0.016
Some college or more	4%	--	4%	--	0.933
Married	65%	--	70%	--	0.094
Number of drinks consumed in the past 30 days	16.46	44.51	15.20	39.23	0.680
*Social Context*					
Neighborhood danger and drugs	3.11	1.07	2.77	1.12	<0.001
Advertisement on newspapers/magazines (low exposure)	23%	--	35%	--	<0.001
Advertisement on newspapers/magazines (moderate exposure)	39%	--	35%	--	0.226
Advertisement on newspapers/magazines (high exposure)	39%	--	30%	--	0.016
Advertisement on television (low exposure)	18%	--	28%	--	0.001
Advertisement on newspapers/magazines (low exposure)	32%	--	31%	--	0.894
Advertisement on newspapers/magazines (moderate exposure)	50%	--	41%	--	0.007

^a^ *p*-value of *t*-test for continuous variables and chi-square test for categorical variables.

### 3.2. Negative Binomial Regression under the Homogeneity Assumption

Given the long right tail of the distribution of alcohol consumption (90.48% of youth had 0, 1, or 2 drinks in the past 30 days) and the count nature of the dependent variable (the number of drinks consumed in the past 30 days), we used a negative binomial regression model to estimate the relationship between peer drinking and youth’s consumption of alcoholic drinks controlling for youth demographic, socioeconomic, parental, and neighborhood characteristics. Results of the multivariate negative binomial regression are shown in Table 2. Under the homogeneity assumption, the size of the relationship between peer and youth alcohol consumption was equal among all youth with a coefficient size of 2.40 regardless of their probability of contact with drinking peers. This indicates that youth’s affiliation with peer drinkers was associated with a difference of 2.40 in the log of the expected count of drinks. Alternatively, youth associated with peer drinkers, compared to having no friends who drink, was expected to have a rate 11 times greater (= *e*^2.40^) for own alcohol consumption behavior. These findings are consistent with the claim that youth drinking patterns do not occur in isolation, but are positively associated with peer-drinking patterns [33]. However, the size of the peer-drinking coefficient under the homogeneity assumption may potentially be confounded by selection bias and should be interpreted with caution. More specifically, it may be important to identify possible heterogeneous treatment effects, and examine whether some youth respond differently to the effects of peer drinking compared to others by adopting propensity score methods.

Control variables highlighted the important role of demographic and environmental contexts. Consistent with previous research [34], older age was positively associated with higher frequency of drinks. There was some evidence that being male and parent’s level of alcohol consumption was associated with increased level of youth drinking behavior. Neighborhood risk was positively correlated with greater levels of alcohol consumption. Social processes such as increased exposure to opportunities for unstructured and unsupervised activities in at-risk neighborhoods may have increased the risk of youth’s engagement in problem behaviors [35].

**Table 2:** Relationship between Peer-Drinking and Youth-Drinking under Homogeneity Assumption (*N* = 914).

Variables	Coefficient	Standard Error	Significance
*Peers*			
Peer alcohol consumption	2.40	0.35	***
*Youth*			
Male	0.40	0.23	^†^
Age	0.57	0.09	***
*Family Context*			
Monthly income (unit: 100 Chilean pesos)	−0.47	0.29	
Monthly income (squared)	0.05	0.03	
Less than middle school ^a^	0.04	0.73	
Middle school to less than high school ^a^	−0.39	0.60	
High school ^a^	−0.39	0.59	
Married	−0.04	0.25	
Number of drinks consumed in the past 30 days	0.01	0.00	^†^
*Social Context*			
Neighborhood danger and drugs	1.88	0.66	**
Neighborhood danger and drugs (squared)	−0.27	0.10	**
Advertisement on newspapers/magazines (low exposure) ^b^	−0.34	0.38	
Advertisement on newspapers/magazines (moderate exposure) ^b^	−0.45	0.26	
Advertisement on television (low exposure) ^c^	−0.47	0.40	
Advertisement on television (moderate exposure) ^c^	0.06	0.26	
Constant	−12.10	1.75	***

*** *p* < 0.001; ** *p* < 0.01; * *p* < 0.05; ^†^ *p* < 0.1; ^a^ Reference group is some college or more; ^b^ Reference group is advertisement on newspapers/magazines (high exposure); ^c^ Reference group is advertisement on television (high exposure).

### 3.3. Propensity Score Analysis under the Heterogeneity Assumption

Treatment effects estimated under the homogeneity assumption can be viewed as the weighted average of heterogeneous effects across individuals. Considering the complex nature of the relationship between peer drinking and youth drinking, in which individuals have different levels of susceptibility and responsiveness to peers, relying on a single weighted treatment effect can be misleading and unrealistic [16]. Therefore, we derived a series of parameter estimates assuming heterogeneity in the probability of associating with deviant peers, using propensity score stratification.

First, we computed the propensity scores by estimating an individual’s predicted probability of affiliating with drinking peers based on observed information (Table 3). Age had a positive relationship with youth’s affiliation with peer drinkers. Dangerous neighborhoods and exposure to alcohol advertisements were also associated with a greater chance of having friends who drink.

**Table 3:** Propensity Score Estimation with Probit Regression Model (*N* = 914).

Variables	Coefficient	Standard Error	Significance
*Youth*			
Male	−0.11	0.10	
Age	0.46	0.04	***
*Family Context*			
Monthly income (unit: 100 Chilean pesos)	−0.05	0.12	
Monthly income (squared)	0.01	0.01	
Less than middle school ^a^	0.10	0.33	
Middle school to less than high school ^a^	−0.14	0.28	
High school ^a^	0.17	0.27	
Married	0.01	0.10	
Number of drinks consumed in the past 30 days	0.00	0.00	
*Social Context*			
Neighborhood danger and drugs	0.58	0.24	*
Neighborhood danger and drugs (squared)	−0.07	0.04	^†^
Advertisement on newspapers/magazines (low exposure) ^b^	−0.27	0.14	^†^
Advertisement on newspapers/magazines (moderate exposure) ^b^	−0.01	0.12	
Advertisement on television (low exposure) ^c^	−0.20	0.14	
Advertisement on television (moderate exposure) ^c^	−0.06	0.12	
Constant	−6.79	0.75	***

*** *p* < 0.001; ** *p* < 0.01; * *p* < 0.05; ^†^ *p* < 0.1; ^a^ Reference group is some college or more; ^b^ Reference group is advertisement on newspapers/magazines (high exposure); ^c^ Reference group is advertisement on television (high exposure).

**Figure 1:**
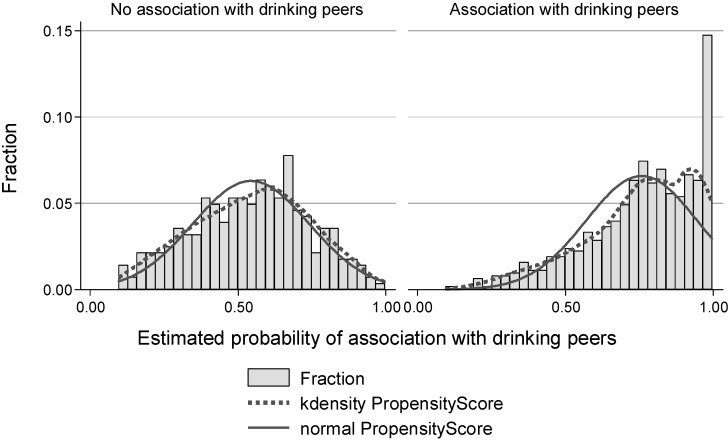
Histogram of Estimated Propensity Score by Association with Peer Drinkers (*N* = 914).

A histogram (Figure 1) shows the relative distribution of estimated propensity scores for youths who associated with peers who drink and youths who associated with peers who do not drink. Among youth who actually had friends who drank, their predicted probability of having drinking friends was high, and *vice versa*. These results were consistent with the way in which the propensity scores were initially constructed [17].

We then stratified our sample into five groups based on these propensity scores and confirmed that the balancing conditions were met for each stratum. The number of observations in each stratum ranged from 108 to 330. Although the overall distributions of propensity scores were different between youth who associated and did not associate with peer drinkers (Figure 1), within each stratum, mean values of the covariates were generally not statistically different between the two groups (Table 4).

**Table 4:** Test of Balance across Propensity Score Strata (*N* = 914).

Peer Alcohol Consumption	Stratum 1			Stratum 2			Stratum 3			Stratum 4			Stratum 5		
	Mean		*p*-Value ^a^	Mean		*p*-Value ^a^	Mean		*p*-Value ^a^	Mean		*p*-Value ^a^	Mean		*p*-Value ^a^
	Yes (*n* = 38)	No (*n* = 70)		Yes (*n* = 84)	No (*n* = 96)		Yes (*n* = 80)	No (*n* = 56)		Yes (*n* = 129)	No (*n* = 31)		Yes (*n* = 300)	No (*n* = 30)	
*Youth*															
Male	0.68	0.56	0.20	0.54	0.52	0.84	0.54	0.66	0.15	0.42	0.48	0.51	0.48	0.43	0.60
Age	12.35	12.40	0.59	13.27	13.22	0.65	13.93	13.85	0.34	14.35	14.43	0.45	15.95	15.21	0.00
*Family Context*															
Monthly income (unit: 100 Chilean pesos)	2.52	2.78	0.26	3.02	3.22	0.41	3.10	2.94	0.50	3.22	2.46	0.02	3.34	3.28	0.85
Less than middle school	0.05	0.03	0.53	0.13	0.03	0.01	0.04	0.11	0.11	0.06	0.06	0.96	0.04	0.13	0.03
Middle school to less than high school	0.53	0.63	0.30	0.37	0.40	0.71	0.40	0.34	0.47	0.34	0.48	0.14	0.31	0.23	0.38
High school	0.39	0.31	0.40	0.48	0.53	0.46	0.53	0.50	0.77	0.56	0.45	0.29	0.61	0.60	0.94
Some college or more	0.03	0.03	0.95	0.02	0.04	0.51	0.04	0.05	0.65	0.04	0.00	0.27	0.04	0.03	0.86
Married	0.76	0.81	0.53	0.73	0.72	0.91	0.63	0.61	0.83	0.68	0.58	0.28	0.60	0.70	0.29
Number of drinks consumed in the past 30 days	19.68	10.60	0.06	22.08	17.00	0.52	10.31	19.75	0.11	11.60	7.65	0.43	18.21	19.47	0.90
*Social Context*															
Neighborhood danger and drugs	2.48	2.40	0.71	2.75	2.78	0.82	2.80	2.93	0.51	3.03	2.95	0.68	3.41	3.13	0.16
Advertisement on newspapers/magazines (low exposure)	0.37	0.57	0.04	0.45	0.35	0.18	0.25	0.18	0.32	0.22	0.19	0.77	0.14	0.30	0.02
Advertisement on newspapers/magazines (moderate exposure)	0.37	0.24	0.17	0.31	0.34	0.63	0.36	0.45	0.33	0.37	0.42	0.63	0.43	0.33	0.32
Advertisement on newspapers/magazines (high exposure)	0.26	0.19	0.35	0.24	0.30	0.34	0.39	0.38	0.88	0.41	0.39	0.81	0.43	0.37	0.48
Advertisement on television (low exposure)	0.34	0.43	0.38	0.26	0.32	0.37	0.16	0.21	0.44	0.24	0.10	0.08	0.12	0.13	0.83
Advertisement on television (moderate exposure)	0.24	0.27	0.70	0.39	0.30	0.20	0.36	0.36	0.95	0.26	0.29	0.76	0.31	0.37	0.55
Advertisement on television (high exposure)	0.42	0.30	0.21	0.35	0.38	0.68	0.48	0.43	0.59	0.50	0.61	0.24	0.57	0.50	0.48
Observations (*n*)	38	70	--	84	96	--	80	56	--	129	31	--	300	30	--

^a^: *p*-value of *t*-test for continuous variables and chi-square test for categorical variables.

**Figure 2:**
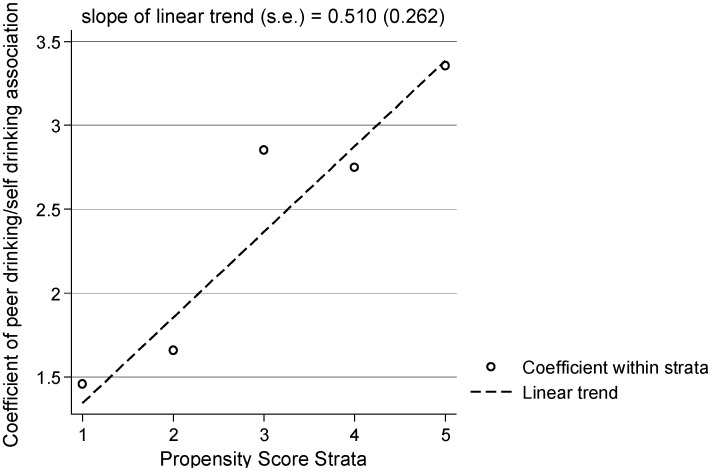
Graphical Representation of Heterogeneous Size of Peer-Drinking and Youth-Drinking Relationship (*N* = 914).

After confirming that the two groups were reasonably homogeneous within each stratum we estimated the differential associations between peer drinking and youth drinking. For each stratum, we estimated the relationship between peer drinking and youth drinking using a negative binomial regression model. We found differences in the associations between peer and youth alcohol consumption patterns after accounting for some level of variability. Table 5 summarizes Level-1 and Level-2 results. Comparison of Level-1 slopes indicated that youths who were most likely to socialize with drinking friends (stratum 5) showed a larger response to peer drinkers (β = 3.36, *p* < 0.001) relative to youths who were least likely (stratum 1; β = 1.46, *p* = 0.198). These values provide a more accurate range of parameters of the relationship between peer and youth drinking, in contrast with the single relationship parameter of 2.40 under the homogeneity assumption. The Level-2 slope of 0.51, trending toward significance (*p* = 0.052), suggested that the size of the association between peer and youth drinking may increase linearly across strata (Figure 2). When assuming heterogeneous relationship sizes, the role of peers on youth drinking increased, with greater levels of risk of associating with friends who drink. Evidence of such heterogeneous relationship sizes substantiated the idea that youth alcohol consumption proliferates when youth are more likely to socialize with peer drinkers.

**Table 5:** Relationship between Peer-Drinking and Youth-Drinking under Heterogeneity Assumption.

Stratum	Coefficient	Standard Error	*p*-Value	Significance	Observations
**Level-1**					
1	1.46	1.13	0.198		108
2	1.66	0.66	0.012	*	180
3	2.85	0.92	0.002	***	136
4	2.75	0.90	0.002	***	160
5	3.36	0.70	0.000	***	330
**Level-2**					
Slope	0.51	0.26	0.052	^†^	914
Constant	0.83	0.91	0.361		

*** *p* < 0.001; ** *p* < 0.01; * *p* < 0.05; ^†^ *p* < 0.1.

Finally, we also investigated a rich set of behavioral measures to check the plausibility of invoking the strongly ignorable treatment assumption (Table 6). Behavioral measures can reflect latent dispositions of individuals who are more susceptible to negative peer influence and are likely to actively seek out associations with similar friends. Rule-breaking behavior, aggression, and risk-taking behavior have been found to characterize antisocial youth [1]. Table 6 illustrates that individuals with the lowest probability of engaging with drinking peers (stratum 1) did in fact have significantly lower problem behavior scores (*p* < 0.001) relative to youth with the highest estimated propensity scores (stratum 5). There was no variability, however, in self-esteem—a finding different from some studies that have highlighted its protective nature [11]. The self-esteem measure employed in our study provides a less-representative understanding of the dispositions of adolescents who increase their exposure to other peers.

**Table 6:** Evidence of Selection-Based Stratification (*N* = 914).

Variables	Stratum 1	Stratum 2	Stratum 3	Stratum 4	Stratum 5	(1)–(5)
	Mean	Mean	Mean	Mean	Mean	*p*-Value ^a^
Rule-Breaking	4.26	4.46	4.36	5.01	5.90	<0.001
Aggression	6.44	7.65	7.69	9.26	8.90	<0.001
Risk-Taking	15.61	16.16	16.59	17.03	17.71	<0.001
Self-esteem and Satisfaction	28.19	28.36	28.45	26.86	28.02	0.746

^a^ *p*-value of the *t*-test between Stratum 1 and Stratum 5.

## 4. Conclusions

This study found that peers’ drinking behavior and youth’s own drinking behavior were significantly linked. Furthermore, the positive relationship between peer influence and the probability of having friends who consume alcohol suggests that the harmful role of drinking friends on an individual’s own drinking behavior may be exacerbated among youth who already have a high probability of socializing with them. In addition, our findings provide evidence that youth with greater aggressive, rule-breaking, and risk-taking tendencies—dispositions that have previously been documented as factors that increase susceptibility to negative peer-effects [1]—had greater risk of consuming alcohol when in contact with other at-risk peers. Consistent with prior research of adolescent alcohol consumption with propensity score stratification [36], methodologically, this study demonstrates that computing a single weighted-average estimate for peer drinking (e.g., standard analytic procedures such as ordinary least squares and logistic regressions) underestimates the detrimental role of peers, particularly among at-risk youths. On the other hand, a single estimate tends to overestimate the role of peers among youths who are less susceptible to peer socialization processes.

Although examination of heterogeneous relationship sizes may provide a better understanding of peer and youth drinking patterns in comparison to a single peer-drinking coefficient, there are limitations to our findings. At a minimum, longitudinal data would be necessary in order to establish a temporal order in peer and youth alcohol consumption patterns. With propensity score stratification, we have estimated the heterogeneous magnitudes of the associations between peer and youth drinking, accounting for individual variability in the response to peer drinkers. Furthermore, through auxiliary analyses, we have revealed that youth show differential levels of risk-taking, aggression, and rule-breaking behaviors by stratum. This finding suggests that the five strata produced by our estimated propensity scores represent the systematically different responses to patterns of peer drinking, and support the plausibility of invoking the strongly ignorable treatment assignment assumption regarding some behavioral measures. Finally, the current study assumed a linear pattern in the size of the association between peer and youth drinking. It may be possible, however, that there exists a non-linear relationship represented by a steeper slope as the propensity to associate with peer-drinkers increases. The next line of research would involve relaxing the linear assumption and fitting a non-parametric model [37].

Notwithstanding these limitations, our findings shed light on the potential consequences of some commonly used intervention strategies for responding to youth substance use and delinquency. In using propensity score stratification methods, results indicated that the link between peer-drinking and youth’s own drinking may be exacerbated particularly among youth who have characteristics that place them with higher probability to engage with friends who consume alcohol. This information yields substantively important practical implications as it helps identify whether, and to what extent, a treatment (*i.e.*, “association with peer-drinkers”) may have different effects according to youth’s selection into the treatment [37]. For example, there has been an increasing concern that interventions that separate problem youth from mainstream youth and aggregate them (e.g., alternative schools, therapy groups, and juvenile justice facilities) may in fact increase opportunities for detrimental peer effects [1,21]. The heterogeneous role of peer drinking in youth alcohol consumption patterns identified in this study provides further support for the validity of such concerns. Furthermore, our results substantiate the potential benefits of intervention programs that encourage at-risk youth to develop relationships with prosocial peers and networks.
