# *Addendum:* Han, Y.; Grogan-Kaylor, A.; Delva, J.; Xie, Y. Estimating the Heterogeneous Relationship Between Peer Drinking and Youth Alcohol Consumption in Chile Using Propensity Score Stratification. *Int. J. Environ. Res. Public Health* 2014, *11*, 11879–11897

**DOI:** 10.3390/ijerph120201649

**Published:** 2015-01-30

**Authors:** Yoonsun Han, Andrew Grogan-Kaylor, Jorge Delva, Yu Xie

**Affiliations:** 1 Department of Child Psychology and Education, Sungkyunkwan University, 25-2 Sungkyunkwan-ro, Jongno-gu, Seoul 110-745, Korea; 2 School of Social Work, University of Michigan, 1080 South University Avenue, Ann Arbor, MI 48109, USA; E-Mails: agrogan@umich.edu (A.G.-K.); jdelva@umich.edu (J.D.); 3 Institute for Social Research, University of Michigan, 426 Thompson Street, Ann Arbor, MI 48109, USA; E-Mail: yuxie@umich.edu

The authors wish to update the Acknowledgments in their paper published in *International Journal of Environmental Research and Public Health* [1], doi:10.3390/ijerph111111879, website: http://www.mdpi.com/1660-4601/11/11/11879.
